# Immature dendritic cell-targeting mRNA vaccine expressing PfCSP enhances protective immune responses against *Plasmodium* liver infection

**DOI:** 10.21203/rs.3.rs-4656309/v1

**Published:** 2024-07-09

**Authors:** Sean Yanik, Varsha Venkatesh, James T. Gordy, Mohamad Gabriel-Alameh, Jacob Meza, Yangchen Li, Elizabeth Glass, Yevel Flores-Garcia, Ying Tam, Nattawat Chaiyawong, Deepti Sarkar, Drew Weissman, Richard Markham, Prakash Srinivasan

**Affiliations:** 1 Department of Molecular Microbiology and Immunology, Johns Hopkins School of Public Health, Baltimore, MD, 21205, USA.; 2 The Johns Hopkins Malaria Research Institute, Baltimore, MD, 21205, USA.; 3 Penn Institute for RNA Innovation, University of Pennsylvania, Philadelphia, PA 19104; 4 Acuitas Therapeutics, Vancouver, BC, Canada.

## Abstract

Resurgence in malaria has been noted in 2022 with 249 million clinical cases resulting in 608,000 deaths, mostly in children under five. Two vaccines, RTS, S, and more recently R21, targeting the circumsporozoite protein (CSP) are recommended by the WHO but are not yet widely available. Strong humoral responses to neutralize sporozoites before they can infect the hepatocytes are important for vaccine-mediated protection. Suboptimal protection conferred by these first-generation vaccines highlight the need for approaches to improve vaccine-induced immune responses. With the recent success of mRNA-LNP vaccines against COVID-19, there is growing interest in leveraging this approach to enhance malaria vaccines. Here, we present the development of a novel chemokine fusion mRNA vaccine aimed at boosting immune responses to PfCSP by targeting the immunogen to immature dendritic cells (iDC). Vaccination of mice with mRNA encoding full-length CSP fused to macrophage inflammatory protein 3 alpha (MIP3α) encapsulated within lipid nanoparticles (LNP) elicited robust CD4+ T cell responses and enhanced antibody titers against NANP repeat epitopes compared to a conventional CSP mRNA-LNP vaccine. Importantly, the CSP-MIP3α fusion vaccine provided significantly greater protection against liver infection upon challenge with *P. berghei* PfCSP transgenic sporozoites. This enhanced protection was associated with multifunctional CD4+ T cells levels and anti-NANP repeat titers. This study highlights the potential to augment immune responses to PfCSP through iDC targeting and bolster protection against malaria liver infection.

## Introduction

Prevention of malaria is currently a global health priority, with half of the world’s population living in at-risk areas and inflicting a staggering 249 million clinical cases and 608,000 deaths in 2022 alone and, by some estimates, resulted in over 150 million deaths since the 1890s (1, 2). The first WHO authorized malaria vaccine, RTS,S, reduces clinical malaria by ~30% over a four year period (3) and is beginning to be made available in some African countries. R21, an RTS,S-related malaria vaccine with similar short-term efficacy was also recently authorized by the WHO (4, 5).

Early studies on *Plasmodium spp*. sporozoites showed that immunization with irradiated sporozoites provided sterile protection in mice (6, 7), monkeys (8), and humans (9–13). Notably, protection was temporally correlated with antibody titers against circumsporozoite protein (CSP) (14), and most of the neutralizing antibodies targeted CSP (15). CSP is among the most highly expressed sporozoite proteins (16, 17) and has a critical functional role in invasion of human hepatocytes (18, 19) and parasite development within mosquito salivary glands (20). Furthermore, monoclonal antibodies against CSP prevent hepatocyte invasion by *Plasmodium spp.* (21–23) and antibodies induced by immunization with CSP may provide sterile protection when reaching high anti-CSP titers (5, 24). CSP expression is confined to the sporozoite stage. Sporozoites are deposited in the skin during a mosquito bite before finding their way through the blood to infect the hepatocyte after which the parasite is immune to inhibition by anti-CSP antibodies (25). Therefore, CSP targeting vaccines must generate a robust immune response capable of neutralizing all sporozoites in a time frame on the order of minutes. For these reasons, conventional approaches to CSP vaccines have been unable to generate the robust immune responses necessary for consistent human protection.

In the last decade, scientific advances in nucleic acid vaccines have been applied to preclinical testing of novel malaria vaccines, including DNA vaccines (26), self-amplifying RNA replicon constructs (27) and lipid nanoparticle (LNP) encapsulated mRNA formulations (28, 29). Unlike protein subunit vaccines, RNA vaccines induce both strong B and T cell responses, critical for sterile immunity (30–33). They are also strong inducers of T follicular helper cell responses, associated with long-lasting malaria immunity (34). Vaccine designs that enhance dendritic cell targeting and activation offer enticing mechanisms for enhancing antibody titers (35, 36). Recent efforts fusing target antigens to cytokines, such as granulocyte-macrophage colony-stimulating factor (37), macrophage inflammatory protein 1 alpha (38), and macrophage inflammatory protein 3 alpha (MIP3α) have successfully enhanced vaccine-induced immune responses (39). MIP3α, also known as chemokine ligand 20 (CCL20), is expressed on immature dendritic cells (40) and acts as a strong chemotactic agent of dendritic cells (41–43). Co-expression of MIP3α with a target antigen, may enhance the immune response to melanoma tumors (44, 45), colon adenocarcinoma (44), and HIV (46). Vaccine fusion to MIP3α elicits greater protection by attracting immune cells to site of immunization and subsequently targeting the antigen to CCR6 on surface of immature dendritic cells. Recently, a DNA vaccine encoding CSP fused to MIP3α provided greater protection in mice than CSP alone (26). Similarly, a protein subunit vaccine using CSP fused to MIP3α conferred superior protection against *Plasmodium spp.* in mice (47) and generated higher anti-CSP antibody titers in nonhuman primates than CSP alone (39). However, low protein expression and concerns over potential insertional mutagenesis have so far hindered development of other DNA vaccines for human use (48). In this study, we sought to combine novel advances in RNA vaccines, lipid nanoparticles, and dendritic cell targeting to create a pre-erythrocytic malaria vaccine that elicits strong cellular and humoral responses targeting CSP.

## Methods

### Production of mRNA-LNP formulations

A codon optimized 3D7 PfCSP sequence (NCBI Reference Sequence: XM_001351086.1) coding for amino acids 22–374 was used in this study. The human tissue plasminogen activator (tPA) signal sequence was fused to the N-terminus of the PfCSP construct (49). The tPA signal sequence and human MIP3α gene were fused to N-terminus of PfCSP to create a chemokine fusion construct. A C-terminus myc tag was added to both constructs to help detect the expressed protein. The DNA sequence was synthesized and cloned into a RNA production plasmid as previously described (50). Capped, m1ψ-5’-triphosphate RNA with a 101-nucleotide poly(A) tail was synthesized and encapsulated in LNP as described (50). RNA was purified using cellulose purification as previously described (51). Purity was confirmed via gel electrophoresis, and transcripts were stored at −20°C until encapsulation in LNP. LNP encapsulation of mRNA was done by rapidly mixing an acidic aqueous solution of cellulose purified mRNA with an ethanolic mixture of cationic lipid, cholesterol, polyethylene glycol-lipid, and phosphatidylcholine (Acuitas Therapeutics) as described (52). The ionizable lipid and LNP composition are described in US patent US10,221,127. The mean hydrodynamic diameter of mRNA-LNPs was ~80 nm with a polydispersity index of <0.1 and an encapsulation efficiency of ~95%. Gel electrophoresis with a 2.5% formaldehyde gel was utilized to confirm the purity of both mRNA-LNP formulations. Finally, LNP-encapsulated mRNA was stored at −80°C at a concentration of 1mg/ml until further use.

### Peptide Synthesis

The NANP6 peptides used in this study were synthesized by LifeTein (South Plainfield, NJ). High-performance liquid chromatography (HPLC) was used to assess purity and demonstrated the NANP6 peptides were over 95% pure.

### Protein Expression and Purification

A *E.coli* codon optimized full length PfCSP vaccine sequence was cloned into a pET47b vector and isolated by 6x His tag purification on an IMAC column as previously described (47).

### Western Blot

HEK293T cells (Human Embryonic Kidney cells) were grown at 37°C and 5% CO_2_ using DMEM media with 10% FBS and 1mg/ml of penicillin and streptomycin. HEK 293T cells were plated at a seeding density of 200,000 cells/well in a 6 well tissue culture treated plate (Avantor). Plates were kept at 37°C with 5% CO_2_ until the culture reached approximately 60% confluency (24–48hrs). The cells were transfected with 2μg/well of either the CSP mRNA-LNP formulation or the MIP3α-CSP mRNA-LNP formulation and returned to the 37°C and 5% CO_2_ incubator. After 48hrs, protein from cell lysates and culture supernatants were run under reducing conditions on a 4–12% Bis-tris SDS-PAGE gel (Invitrogen) and transferred to nitrocellulose membranes. Protein expression was determined by probing the blot with mouse anti-myc primary antibody (1:1000, Genscript) in blocking buffer and detected using goat anti-mouse IgG (H+L) + HRP (1:2000, Cell Signaling Technologies). Membranes were developed with SuperSignal chemiluminescence reagent (Thermofisher Scientific, Waltham, MA) and viewed under a UV transilluminator (Syngene G Box).

### Immunofluorescence assay (IFA)

HEK 293T cells were plated at a seeding density of 200,000 cells/well, topped with sterile coverslips (12 mm × 12 mm), and grown at 37°C and 5% CO_2_ for 48hrs. Once 60% confluency was reached, cells were transfected with 2μg/well of the CSP mRNA-LNP formulation, 2μg/well of the MIP3α-CSP mRNA-LNP formulation, or sterile PBS as a non-transfected control and returned to the 37°C and 5% CO_2_ incubator. After 48hrs, each well was fixed for 30 min at RT with 2.5% formaldehyde & 0.05% glutaraldehyde in PBS, washed 3x with 2ml PBS, and then blocked with 2% BSA and 0.1% Triton X-100 in PBS for 1hr at RT. Protein was detected using mouse anti-myc antibody (1:1000, Genscript) in blocking buffer for 1hr followed by goat-anti mouse Alex Fluor 588 antibody (1:500, Thermofischer Scientific). Images were captured using a LEICA DMi8 microscope as a z stack, deconvolved with LAS X.

### Ethics statement

All animal procedures followed the Guide for the Care and Use of Laboratory Animals of the National Institutes of Health and were approved by the Johns Hopkins Animal Care and Use Committee approved protocol (MO22H289). Mice were housed at the Johns Hopkins Bloomberg School of Public Health animal facility were conditions are maintained at 40–60% relative humidity and 68–79 F, with at least ten room air changes per hour and a 14/10-hour light/dark cycle. No animals or data points were excluded from analyses.

### Animals, Antigen dose, and Immunizations

Six to seven-week-old female C57BL/6J mice (The Jackson Laboratory) were used in vaccination studies and were randomly assigned to different vaccination groups. Sample sizes were based on previous published data (53) for testing CSP-targeting vaccines that would permit differentiating between different groups. Following vaccinations, mice groups were blinded to the researcher prior to *in vivo* evaluations and un-blinded at the end of study. The order of vaccinations and challenge were randomized to avoid any confounders. Mice were anesthetized under isoflurane prior to vaccinations and IVIS imaging. Terminal bleeds and spleen collection were performed following euthanasia by CO2. Toe pinch was used to determine sufficiency followed by cervical doislocation to ensure proper euthanasia. For small volume blood collection from tail snip and parasite challenge studies, mice were held in a restrainer and placed gently back in the respective cages after these minor procedures and monitored for atleast 48h. All protocols used in animals studies were approved by Johns Hopkins Animal Care and Use Committee (ACUC), under protocol MO22H289.

Standard dose: Groups of mice (n=5/group) received 10μg of rCSP, LNP-CSP mRNA or LNP-Mip3α-CSP mRNA diluted in 50μl sterile 1X phosphate buffered saline (PBS, pH 7.4) intramuscularly three times at 2-week intervals. Sera was collected prior to subsequent immunizations or parasite challenge for analysis.

Dose de-escalation and delayed 2^nd^ boost: Mice (n=7/group) received 25μg, 15μg and 5μg of LNP-CSP mRNA or LNP-Mip3α-CSP mRNA diluted in 50μl sterile 1X phosphate buffered saline (PBS, pH 7.4) intramuscularly at 0, 2 and 6-week intervals respectively. Sera was collected prior to subsequent immunizations or parasite challenge for analysis.

### Sporozoite challenge

Transgenic *P. berghei* sporozoites expressing *P. falciparum* CSP and luciferase (Pb PfCSP-luc) were used in this study (54). Vaccine induced protection was assessed by challenging immunized mice with 3000 Pb PfCSP-luc sporozoites two weeks after the last immunization. Forty hours after the challenge, mice were injected intraperitoneally with 100μL of D-luciferin (30mg/mL, PerkinElmer), anesthetized with isoflurane, and imaged on an IVIS^®^ Spectrum *in vivo* Imaging System (Perkin Elmer). Bioluminescence measurements of mice abdomens were taken and normalized to those of unvaccinated mice challenged with Pb PfCSP-luc sporozoites.

### ELISAs

ELISAs were performed as described (55) with the following modifications. Immulon 4HBX flat bottom 96-well plates (Thermofisher Scientific) were coated with either rCSP (0.5 μg/ml) or NANP6 peptide (2 μg/ml) and incubated overnight at 4°C. Plates were blocked with 2% milk in PBS for 2h at room temperature (RT). Serum was serially diluted and added to the plates for 2h at RT. Following washing with 1xPBS + 0.1% Tween20, goat anti-mouse HRP secondary antibody (1:2000, Cell Signaling, 7076S) was added for 2h, washed and developed with (BioFx TMB, Surmodics) for 10min. The reaction was stopped with (BioFx 450nm Liquid Nova-Stop Solution, Surmodics). Plates were read at 450nm using a BMG Fluro star omega plate reader, and end point titers were then calculated.

### IgG isotyping

IgG1 and IgG2a isotyping was performed for CSP and MIP3α-CSP groups only from experiment 2. IgG isotypes were calculated using the above ELISA protocol albeit with the following modifications. For the IgG1 study, a starting 1:2000 primary dilution of sera was used and a 1:2000 dilution of the secondary antibody goat anti-mouse IgG1 was used (Invitrogen, PA1-74421). For the IgG2a study, a starting 1:2000 primary dilution of sera was used and a 1:500 dilution of the secondary antibody goat anti-mouse IgG1 was used (Invitrogen, PA1-74421).

### Avidity assay

Duplicate plates were coated with either rCSP or the NANP6 peptide at 0.5μg/ml and 2μg/ml respectively. Serially diluted sera from each mouse were applied to wells for 2h at RT. After washing the excess primary antibody, 100μl of 1x PBS was added to one plate while the other received 100 μl of 5M urea (the chaotropic agent) in PBS. Both plates were then incubated for 30min at RT. The plates were then washed 3x with 0.1% Tween20 in PBS before addition of the secondary antibody (1:2000 HRP goat anti-mouse (Cell Signaling, #7076S) or 1:250 HRP goat anti-mouse (Invitrogen, #32430). Avidity indices were calculated using the following equation ((Titer, dilution OD=1 in 5M urea)/(Titer, dilution OD=1 in PBS)*100).

### Lymphocyte Isolation

Under sterile conditions, mouse spleens were harvested and placed in 1x PBS on ice. Spleens were ground gently with a pestle over a 70μM mesh filter into 50mL conical tubes and immediately centrifuged at 300g for 10min at 4°C. The supernatant was removed, and the pellet was fully resuspended using 1mL ACK lysis buffer (Quality Biological, Gaithersburg, MD) and incubated at room temperature (RT) for 3–4min. To stop cell lysis, cells were diluted with 20–30mL of cold 1x PBS and were then pelleted at 300g for 10min 4°C. After another resuspension in 10mL cold 1x PBS and centrifugation under the same conditions, the supernatant was removed, and the pellet was resuspended in 4mL freezing medium (90% FBS, 10% DMSO). Each pellet was then aliquoted into 4 tubes for cryostorage using isopropanol cooling containers (Mr. Frosty, ThermoFisher Scientific, Waltham, MA). Tubes were stored at −80°C for at least 4h and then moved to −150°C.

### Intracellular Cytokine Staining and Flow Cytometry

Cryopreserved cells were recovered by thawing briefly in a water bath at 37°C and then diluted slowly to 10mL with warm complete media (RPMI, 10%FBS, 1x antibiotics, 20mM HEPES, 1% sodium pyruvate, 1% non-essential amino acids, and 1% L-glutamine). Cells were spun for 7min at 250g and RT and then resuspended in a smaller volume of warm media to obtain a final concentration of 5 × 10^5^ − 5 × 10^6^ viable cells/well in 200μL complete media. The cells were then rested in a 5% CO_2_ incubator at 37°C for 4–8h. Samples were stimulated in duplicate wells with 1μg of purified crude CSP identical to the vaccine sequence at 37°C for 16h. Pools of groups were stimulated for 16h with HA peptide (JHMI Synthesis and Sequencing Facility) for the negative control or for 4h with Cell Activation Cocktail with Brefeldin A (Biolegend) for the positive control. During the final 4h, cells were incubated with Brefeldin-A and costimulatory antibodies anti-CD28 and anti-CD49d (Biolegend Cat. Nos 420601, 102116, and 103629). After stimulation, cells were transferred to a 96-well V-bottom plate. After centrifugation at 300g for 5min at RT, cells were washed with 150μL FACS buffer (0.5% Bovine serum albumin (Sigma-Aldrich, St. Louis, MO) in 1x sterile PBS) and pelleted again. Cells were stained with 100μL/well Live/Dead (L/D) stain (1:1000 dilution in 1x sterile PBS) for 30min at RT in the dark (LIVE/DEAD Fixable Near-IR Dead Cell Stain Kit, ThermoFisher Scientific). Cells were pelleted and resuspended in 150μL FACS buffer and washed. Following the L/D stain, 50μL 2% Fc block (TruStain FcX, Biolegend Cat. No 101320) were added to each well and incubated in the dark for 15min on ice. After centrifugation, cells were stained in the dark for 20min with an anti-mouse monoclonal-antibody (mAb) cocktail (50μL per well, diluted in FACS buffer), including 1:500 FITC conjugated anti-CD4 (Biolegend Cat. No 100405), 1:200 PercPCy5.5 conjugated anti-CD3 (Biolegend Cat. No 100217), and 1:200 Alexa Fluor 700 conjugated anti-CD8 (Biolegend Cat. No. 155022). After centrifuging and resuspending in 50μL Fixation buffer (Cyto-Fast Fix/Perm Buffer Set, Biolegend, Cat No 426803), cells were then incubated in the dark at 4°C overnight. For intracellular cytokine staining, intracellular anti-mouse mAb cocktail (50μL per well, diluted in 1x Cyto-Fast Perm buffer) was used to stain the cells in the dark at RT for 20min. The cocktail includes 1:500 PECy7-conjugated anti-TNFα (Biolegend Cat. No. 506323), 1:100 PE conjugated anti-IL2 (Biolegend Cat No. 503808),1:100 APC-conjugated anti-IFNγ (Biolegend Cat. No. 505809). 100μL Cyto-Fast Perm buffer was added to each well for centrifugation at 400g at RT for 5min. Cells were then washed with FACS buffer and resuspended with 150μL 1xPBS and read on the Attune^™^ NxT flow cytometer (Thermo Fisher Scientific, Waltham, MA). Flow data were analyzed using Flow Jo software (FlowJo 10.8.1, LLC Ashland, OR). Gates were formed using negative stimulation and Full-minus-one staining controls. Averages of the duplicate wells are presented.

### Statistics

For T cell assays, cytokine data was analyzed using ordinary one-way ANOVAs with Tukey’s test for multiple comparisons. Correlation analyses of T cell assays utilized Spearman’s rank correlation coefficient. Differences in antibody titers were compared using two-tailed unpaired t tests (for comparison of 2 groups) and ordinary one-way ANOVAs with Tukey’s test for multiple comparisons (for comparison of 3 groups). In both challenge studies, luminescence values between groups were compared using ordinary one-way ANOVAs with Tukey’s test for multiple comparisons. Percent inhibition between groups was compared using two-tailed unpaired t tests and calculated relative to naïve, sporozoite challenged mice. Prism 10 software was used for analysis, and data was maintained in Microsoft Excel.

## Results

### Characterization of CSP and MIP3α-CSP mRNA vaccines

**Figure 1.**
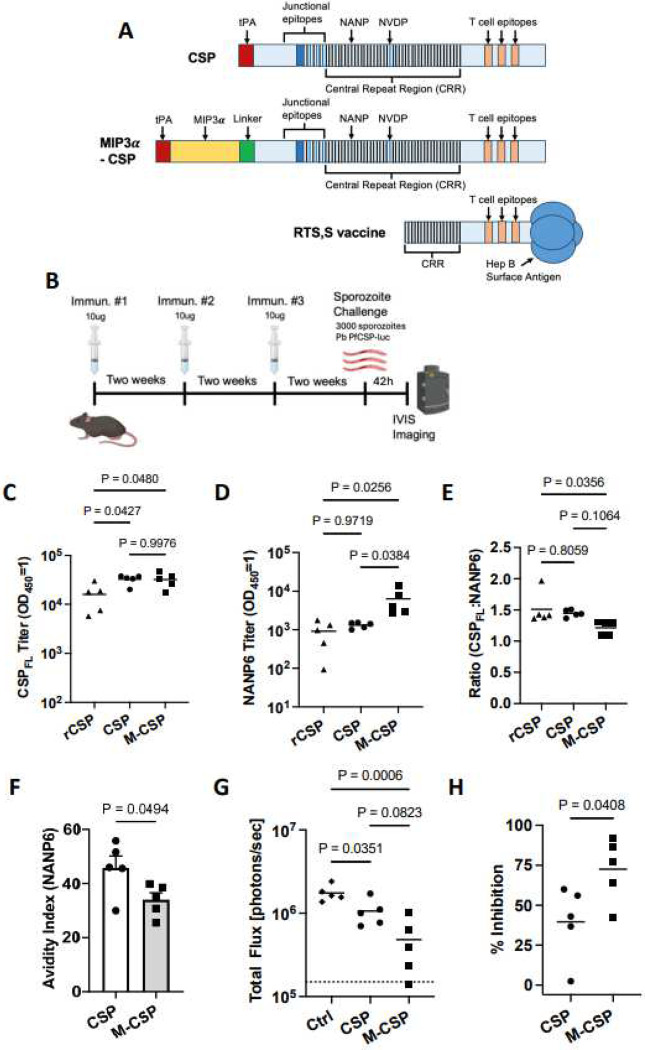
Antibody responses and protection from liver stage infection following standard 3-dose mRNA vaccination. (A) Design of CSP and MIP3α-CSP constructs. Representations of protein sequences for full length CSP, the MIP3α-CSP construct, and the CSP construct used in the approved RTS,S vaccine are shown. Both CSP and MIP3α-CSP sequences contain the CSP C-terminal domain containing important T cell epitopes, CSP junctional region, and central repeat region of CSP with 1/4/38 copies of NPDP/NVDP/NANP respectively. For comparison, the RTS,S construct contains 0/0/19 copies of NPDP/NVDP/NANP respectively. In the MIP3α-CSP construct, the human MIP3α gene was fused to the N-terminus of 3D7 PfCSP gene via a 14 amino acid linker sequence. The tPA signal sequence is located at the N terminus of the MIP3α gene in this construct. (B) In the first challenge study, C57BL/6J mice (n=5/grp) were immunized 3x at two-week intervals with 10ug MIP3α-CSP LNP-mRNA or CSP LNP-mRNA. Two weeks after the 3^rd^ immunization, vaccinated and naïve mice were challenged with 3000 Pb PfCSP-luc intravenously delivered sporozoites. Forty-two h after infection, parasite liver loads, measured by luminescence, were captured. (C-D) Full length recombinant CSP specific (C) and NANP6 peptide specific (D) antibody titers in mouse serum are shown. Endpoint titers (OD_450_ = 1) were used to quantify antibody titers for both groups. Data points are individual mice (n = 5) performed in duplicate, with horizontal lines representing mean values. Ordinary one-way ANOVAs with Tukey’s test for multiple comparisons were performed to compare differences between groups. (E) Titer ratios of anti-full length CSP antibodies to anti-NANP6 antibodies are shown, with horizontal lines representing mean values. An ordinary one-way ANOVA with Tukey’s test for multiple comparisons was performed to compare differences between groups. (F) NANP specific avidity indices are shown for antibodies from CSP and MIP3α-CSP groups. Dots represent avidity indices of individual mice. The avidity index was calculated using the following equation ((OD 1 titer in chaotropic agent)/(OD 1 titer in PBS)*100). A two-tailed unpaired t test was performed to determine p values. Bars represent mean ± SEM. (G) Luminescence values for each mouse in naïve, CSP and MIP3α-CSP groups were calculated in photons/sec, with horizontal lines representing group means. An ordinary one-way ANOVA with Tukey’s test for multiple comparisons was performed to compare differences between groups. (H) Percent inhibition of liver infection was calculated relative to naïve, sporozoite challenged mice. Data is again shown for individual animals, with horizontal lines representing mean values. A two-tailed unpaired t test was used to compare groups.

PfCSP (amino acids 22–374) and a second construct with a N-terminal MIP3α tag were synthesized and encapsulated in LNP (Fig. 1A). LNP-encapsulated CSP and MIP3α-CSP mRNA were stored at −80C and assessed on a 2.5% formaldehyde agarose gel, confirming the presence of a single band of the expected size (Suppl Figure 1A) and demonstrating the stability of the mRNA in the LNP formulation. Next, HEK293T cells were transfected with CSP and MIP3α-CSP LNP to assess protein synthesis. Culture supernatant and cell lysates were collected 48h post-transfection and assayed by Western Blot using anti-myc antibodies. Both CSP and MIP3α-CSP protein were detected in the cell lysate but not supernatant fractions (Suppl Figure 1B), suggesting the amount of protein in the supernatant was below the limit of detection. Immunofluorescence microscopy (IFA) using anti-myc antibodies also confirmed robust expression of both proteins with strong signal observed within the transfected HEK293T cells (Suppl Figure 1C).

### LNP containing MIP3α-CSP mRNA induce significantly higher anti-NANP6 antibody titers compared to CSP mRNA and recombinant CSP

The impact of linking a chemokine to promote immature dendritic cell targeting on vaccine immunogenicity was evaluated by immunizing C57Bl/6J mice with the two mRNA-LNP formulations (10μg/mouse) 3x at two-week intervals. This dose of mRNA was selected because a previous study using the same dose showed partial protection against liver infection (28). Therefore, this dose may allow for a qualitative assessment between the two mRNA groups. A third group of mice was immunized with 10μg of recombinant full length CSP protein (rCSP_FL_) (47), formulated in Addavax adjuvant (Fig. 1B). Humoral responses against rCSP_FL_ and NANP6 peptide (six repeats of NANP) were evaluated by ELISAs in serum collected 2 weeks after the third immunization. Antibody titers against CSP_FL_ were significantly higher in both mRNA vaccines compared to mice that received rCSP_FL_/Addavax, but no difference was observed between CSP and MIP3α-CSP mRNA groups (Fig. 1C). Interestingly, MIP3α-CSP LNP-mRNA vaccinated mice had significantly higher anti-NANP6 peptide antibody titers compared to both rCSP_FL_ and CSP LNP-mRNA vaccinated mice (Fig. 1D). Additionally, the proportion of NANP6 specific antibodies was significantly higher in the MIP3α-CSP LNP-mRNA group than the rCSP_FL_ group but nearly identical between the rCSP_FL_ and CSP LNP-mRNA groups (Fig. 1E). Next, we tested the strength of antibody binding to NANP6 peptide using an avidity ELISA. Interestingly, despite higher levels of overall anti-NANP6 antibodies, MIP3α-CSP LNP-mRNA vaccinated mice had a significantly lower avidity index than CSP LNP-mRNA vaccinated mice (Fig. 1F).

### iDC targeting enhances protection against *Plasmodium* liver stage infection

We used *P. berghei* (Pb) sporozoites expressing PfCSP in place of PbCSP and luciferase (Pb PfCSP-luc) to assess the ability of anti-CSP antibodies to prevent sporozoite infection of hepatocytes (56). Mice previously immunized 3x with 10μg of either MIP3α-CSP or CSP mRNA were challenged intravenously two weeks after the third immunization with 3000 Pb PfCSP-luc sporozoites (Fig. 1B). Naïve mice were used as infection controls. Both CSP and MIP3α-CSP mRNA immunized mice had an overall reduced liver stage burden relative to unvaccinated controls (39.6% and 72.5% respectively) (Fig. 1G & Suppl Figure 2D). Importantly, protection from liver infection was significantly greater in mice immunized with iDC-targeting MIP3α-CSP mRNA compared to CSP mRNA (Fig. 1H).

### Dose de-escalation and delayed boosting enhances cellular and humoral responses induced by MIP3α-CSP mRNA

**Figure 2.**
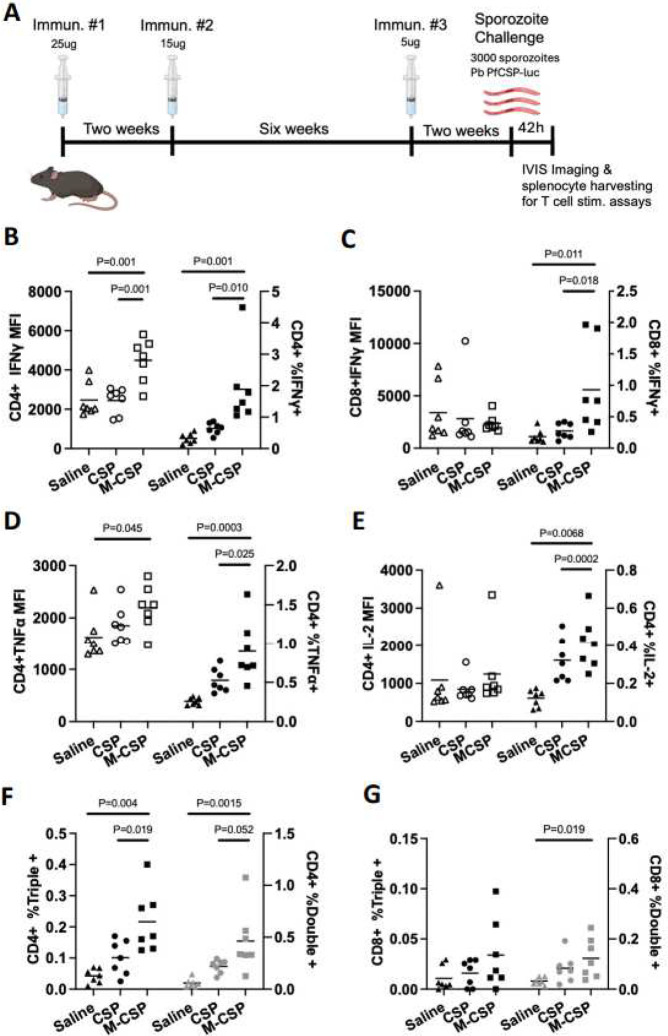
T cell responses in mice following mRNA dose de-escalation and delayed boosting. (A) C57BL/6J mice (n=7/grp) were immunized 3x with 25μg, 15μg, and finally 5μg of MIP3α-CSP LNP-mRNA or CSP LNP-mRNA. In this delayed dosing experiment, the 2^nd^ and 3^rd^ immunizations occurred 2 and 8 weeks after the first immunization respectively. Splenocytes were harvested from each mouse 42h after challenge for usage in T cell stimulation assays. (B-C) Cells were stained, run on flow cytometry, and gated for CD4+ (B) or CD8+ (C) T cells. Median fluorescence intensities of CD4+ IFNγ+ (B) and CD8+ IFNγ+ (C) T cells are shown (left). The percentage of IFN-γ+ cells among CD4+ cells (B) and CD8+ cells (C) are also shown (right). Horizontal lines indicate group means. Ordinary one-way ANOVAs with Tukey’s test for multiple comparisons were performed to compare differences between groups. (D-E) Cells were stained, run on flow cytometry, and gated for CD4+ T cells. Median fluorescence intensities of CD4+ TNFα+ cells (D) and CD4+ IL2+ cells (E) are shown (left). The percentage of TNFα+ (D) or IL2+ cells (E) among CD4+ cells are also shown (right). Horizontal lines indicate group means. Ordinary one-way ANOVAs with Tukey’s test for multiple comparisons were performed to compare differences between groups. (F-G) The percentage of CD4+ or CD8+ (G) T cells that were IFNγ, TNFα, and IL-2 triple positive (left axis) or IFNγ, TNFα double positive are shown (right axis). Horizontal lines indicate group means. Ordinary one-way ANOVAs with Tukey’s test for multiple comparisons were performed to compare differences between groups.

Previous studies with CSP mRNA showed greater antibody titers using a higher mRNA dose (28). Similarly, delayed fractional dose boosting has also been shown to enhance antibody titer and protection from liver infection (28) Therefore, we tested if dose de-escalation combined with a delayed boost may improve vaccine responses. C57Bl/6J mice were vaccinated with 25μg, 15μg and 5μg of either MIP3α-CSP or CSP mRNA in LNP administered at 0, 2 and 6-week intervals (Fig. 2A). Typically, splenic cellular responses are measured in mice immunized in parallel to mice used for challenge studies. Here, we sought to determine the cellular and humoral immune responses from the animals with defined outcomes following challenge. We took advantage of the luciferase expressing parasites allowing for a definitive quantitation of protection outcomes within 42h of challenge. Splenocytes from control, CSP, and MIP3α-CSP mRNA-LNP vaccinated mice were harvested immediately following IVIS imaging. CD4+ T cells play an important role as helpers to promote B cell antibody production and LNP vaccines have been shown to promote strong activation of CD4+ T cell responses (57). CD8+ T cells also play a critical role in the prevention of liver stage infection (30–33). Therefore, we measured the level and proportion of IFNγ, TNFα and IL2 expressing CD4+ and CD8+ T cells by flow cytometry following stimulation with rCSP_FL_ (Suppl Figure 2A–2D).

While the percentage of CD4+ T cells expressing IFNγ, TNFα, or IL2 increased in both mRNA groups relative to control sporozoite challenged mice, the percentage of CD4+ T cells expressing IFNγ, TNFα, or IL2 was significantly higher in the MIP3α-CSP LNP-mRNA group than the CSP LNP-mRNA group (Fig. 2B, 2D and 2E). Furthermore, the mean fluorescence intensity (MFI) of CD4+ T cells expressing IFNγ or TNFα were significantly higher than control mice in the MIP3α-CSP LNP-mRNA group but not CSP LNP-mRNA group (Fig. 2B and 2D). In the case of CD8+ T cells, MIP3α-CSP mRNA enhanced the proportion of cells expressing IFNγ relative to both the control and CSP mRNA groups (Fig. 2C). The role of polyfunctional antigen-specific T cells that express multiple cytokines have been shown to be associated with higher effector function compared to monofunctional T cells and correlate strongly with antibody titers (58–63). Polyfunctional CD4+ T cells that secrete multiple cytokines have been observed in children following both RTS,S vaccination and *P. falciparum* infection (64, 65). Interestingly, mice immunized with iDC-targeting MIP3α-CSP had a significantly higher proportion of CD4+ T cells expressing two or all three cytokines (IFNγ, TNFα, IL2) compared to both control and CSP mRNA groups (Fig. 2F). Likewise compared to control mice, a higher proportion of double positive CD8+ T cells was seen in the MIP3α-CSP LNP-mRNA group but not the CSP LNP-mRNA group (Fig. 2G).

**Figure 3.**
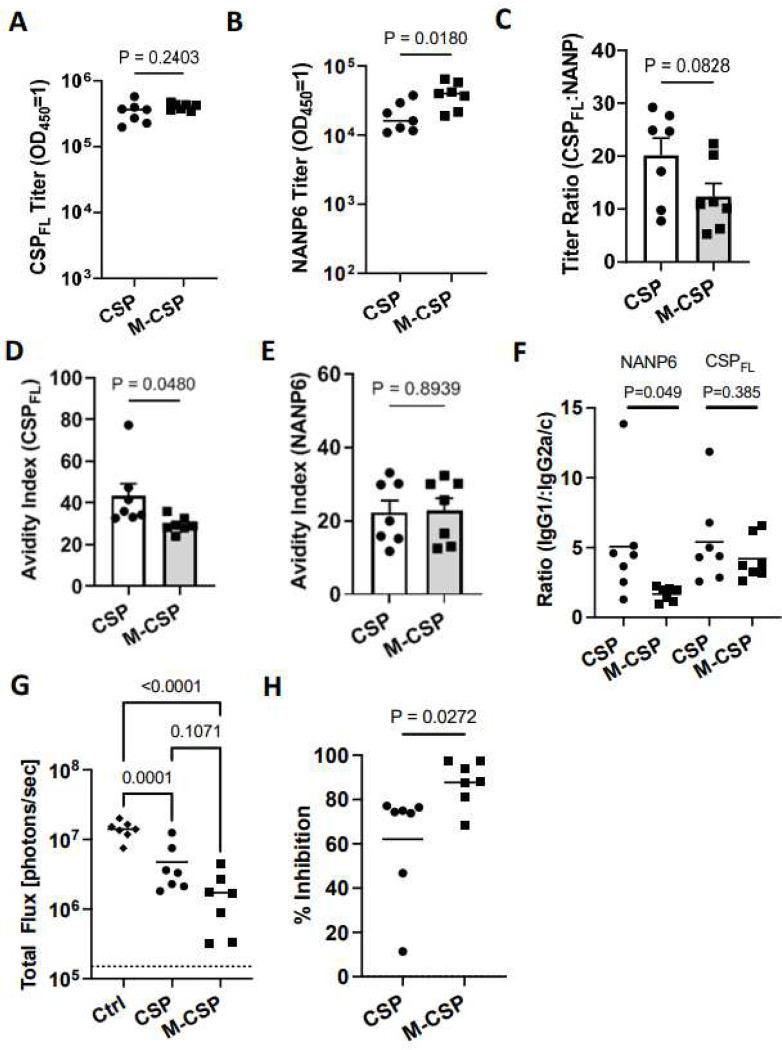
Antibody responses and protection from liver stage infection following mRNA dose de-escalation and delayed boosting. (A) Full length recombinant CSP antibody titer in mice sera. (B) NANP6 peptide-specific antibody titer in mouse sera. Endpoint titers (OD_450_ = 1) were used to quantify antibody titers for both groups. Data points are individual mice (n = 7) performed in duplicate, with horizontal lines representing mean values. Two-tailed unpaired t tests were performed to compare differences between groups. (C) Titer ratios of anti-full length CSP antibodies to anti-NANP6 antibodies are shown, with horizontal lines representing mean values. A two-tailed unpaired t test was performed to compare differences between groups. Data is shown as mean ± SEM with individual points representing individual mice performed in duplicate. (D-E) Full length recombinant CSP specific (D) and NANP6 peptide specific (E) avidity indices are shown for antibodies from CSP and MIP3α-CSP vaccinated mice. Dots represent avidity indices of individual mice performed in duplicate. The avidity index was calculated using the following equation ((OD 1 titer in chaotropic agent)/(OD 1 titer in PBS)*100). Two-tailed unpaired t tests were performed to determine p values. Bars represent mean ± SEM. (F) The IgG1/(IgG2a+IgG2c) ratio of CSP_FL_ specific and NANP6 specific antibodies in CSP and MIP3α-CSP LNP-mRNA vaccinated groups are shown. P values were calculated using two-tailed unpaired t tests. Dots represent the ratio of antibody isotypes of individual mice performed in duplicate, and horizontal bars represent mean values of each group. (G) Luminescence values for each mouse in naïve, CSP and MIP3α-CSP groups were calculated in photons/sec, with horizontal lines representing group means. An ordinary one-way ANOVA with Tukey’s test for multiple comparisons was performed to compare differences between groups. (H) Percent inhibition of liver infection was calculated relative to naïve, sporozoite challenged mice. Data is again shown for individual animals, with horizontal lines representing mean values. A two-tailed unpaired t test was used to compare groups.

Next, we assessed humoral responses in the serum of immunized mice. Antibody titers against rCSP_FL_ and NANP6 were higher in both groups (Fig 3A and 3B) compared to the standard immunization (Fig. 1C and 1D), indicating a positive influence of dose de-escalation and delayed boosting on humoral responses. Interestingly, while no difference in antibody titers to rCSP_FL_ was observed between the groups (Fig. 3A), once again MIP3α-CSP immunized mice had significantly higher anti-NANP6 antibody compared to mice receiving CSP mRNA (Fig. 3B). Additionally, the proportion of NANP6 antibodies, though not significantly different, tended to be higher on average in the MIP3α-CSP mRNA group, (Fig. 3C). Avidity of antibodies induced by MIP3α-CSP was lower against rCSP_FL_ but were similar against NANP6 compared to animals receiving CSP mRNA (Fig. 3D and 3E). Furthermore, while both groups showed similar IgG1:IgG2a/c ratios against rCSP_FL_, NANP6 specific IgG1:IgG2a/c ratios of antibodies induced by MIP3α-CSP were more balanced compared to those induced by CSP (Fig. 3F). The percentage of CD4+ T cells expressing IFNγ correlated significantly with NANP6 titers (Suppl Figure 3A). Similarly, the percentage of triple positive (IFNγ, TNFα and IL2) CD4+ T cells also tended to positively correlate with NANP6 antibody titers (Suppl Figure 3B), suggesting a strong link between cellular and humoral responses induced by the mRNA-LNP vaccines and the immune potentiating effects of MIP3α.

### iDC-targeting MIP3α-CSP LNP-mRNA vaccine confers greater protection than CSP LNP-mRNA vaccine against liver infection in dose de-escalation, delayed-boosting regimen

The effect of dose de-escalation and delayed boosting of vaccines to protect mice against Pb PfCSP-luc sporozoite challenge was evaluated two weeks after the second boost (Fig. 2A). Sporozoite infection of the liver showed enhanced protection of mice in both the groups compared to the first study (CSP mRNA: 62% vs 39.6% and MIP3α-CSP: 88% vs 72.5%) 42h after challenge (Fig. 1H and Fig. 3H). Importantly, once again mice immunized with the iDC-targeting MIP3α-CSP LNP-mRNA vaccine were significantly better protected from liver infection compared to mice immunized with the CSP LNP-mRNA vaccine.

### Correlates of vaccine induced protection

**Figure 4:**
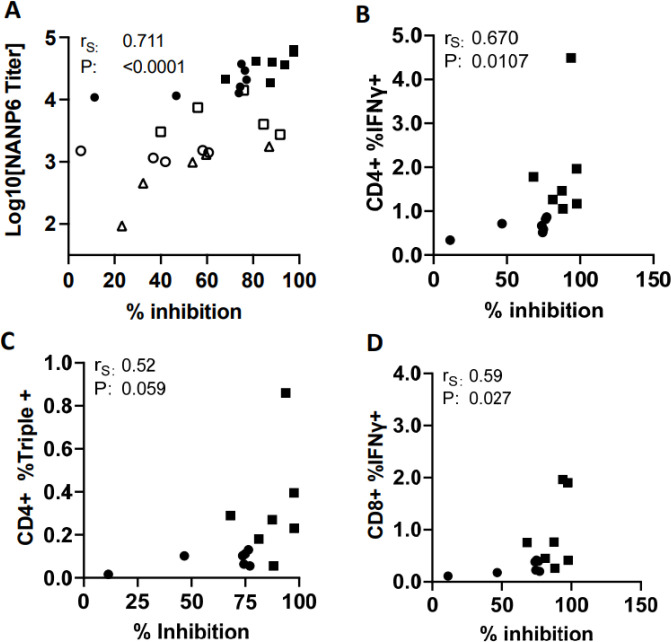
Immunological Correlates of Protection. (A) Relationship between log_10_ NANP6 peptide antibody titers (y-axis) and % inhibition of liver stage parasitemia relative to naïve, sporozoite challenged mice (x-axis). Unfilled and filled symbols represent single mice from challenge studies 1 and 2 (standard regimen and delayed, dose de-escalation regimen) respectively. Squares represent mice from the MIP3α-CSP LNP-mRNA vaccinated group, circles represent mice from the CSP LNP-mRNA vaccinated group, and triangles represent mice from the rCSP_FL_ vaccinated group. P values were calculated using Spearman’s ranked correlation coefficient. (B-D) Relationship between inhibition of liver stage parasitemia (x-axis) and the percentage of IFNγ+ cells among CD4+ cells (B), the percentage of IFNγ, TNFα, and IL-2 triple positive cells among CD4+ cells (C), and the percentage of IFNγ, TNFα, and IL-2 triple positive CD8+ cells (D). Circles represent mice from the CSP vaccinated group, and squares represent mice from the MIP3α-CSP vaccinated group. P values were calculated using

Recent studies demonstrate that antibody titers against the repeat (NANP) region of *P. falciparum* CSP correlate with protection in mice (66–70) and protection from clinical malaria in humans (5). In addition, protection from clinical malaria following RTS,S vaccine administration was associated with the presence of polyfunctional CD4+ T cells (64). Therefore, we sought to examine possible correlations between cellular, humoral responses and protection from liver infection in these mice. This was possible with mice used in the second study since cellular and humoral responses were assessed following IVIS quantitation of liver burden in the corresponding mice. As expected, anti-NANP6 antibody titers correlated positively with anti-CSP_FL_ antibody titers (Suppl Fig. 3C). Importantly, NANP6 antibody titer strongly correlated with protection from liver infection (Fig. 4A), while CSP_FL_ titer also correlated to a lesser extent (Suppl Fig 3D). Similarly, the percentage of CD4+ IFNγ+, CD4+ triple positive (IFNγ, TNFα and IL2), and CD8+ IFNγ+ T cells each positively correlated with reduction in parasite liver burden (Fig. 4B, 4C and 4D).

## Discussion

Despite progress in malaria vaccine development, highlighted by the WHO approval of RTS,S and R21, significant work remains in producing a highly effective vaccine against *P. falciparum* (25, 71). Rapidly spreading resistance to frontline antimalarials (72), a recent rebound in worldwide malaria-related deaths (1), and high population growth in *P. falciparum* endemic countries all intensify the urgency for a highly effective malaria vaccine (73). While targeting the antigen CSP, both the most abundant sporozoite protein (16, 17) and a functionally critical antigen for hepatocyte invasion (18, 19), remains a useful strategy, application of contemporary advances in technology are needed for developing more effective vaccines. The recent success of LNP-encapsulated mRNA vaccine platforms, offers a novel, highly immunogenic (57), and potentially durable (34) strategy for vaccines against malaria. Indeed, full length PfCSP mRNA-LNP vaccines induce protective immunity in preclinical rodent models (28). Novel, chemokine fusion vaccines offer a promising mechanism to enhance immunogenicity and have likewise recently been applied to malaria vaccines with encouraging results (26, 39, 47). In this study, we tested whether immature dendritic cell targeting may further enhance vaccine efficacy. iDC targeting was achieved by using MIP3α, an 8kD (~70 amino acid) chemokine that binds to CCR6 resulting in their activation (74). We engineered a mRNA construct coding for a MIP3α-PfCSP fusion antigen encapsulated within LNP.

Transfection of HEK293T cells confirmed expression of both MIP3α-CSP and CSP proteins by microscopy and western blot analysis. Immunization with the MIP3α-CSP mRNA vaccine resulted in significantly higher antibody titers against the NANP repeat epitope of PfCSP compared to conventional CSP mRNA. We also observed an increase in the proportion of anti-NANP antibodies relative to full length CSP. Greater NANP responses have been associated with an increase in protection from liver stage parasitemia (5, 66, 67). This enhanced humoral response specifically against the functionally critical NANP repeat region of CSP is a promising indicator of the MIP3α-CSP vaccine's ability to elicit potent antibody-mediated immunity against sporozoites.

Mice immunized with MIP3α-CSP mRNA showed a significantly reduced liver stage parasite burden compared to those vaccinated with conventional CSP mRNA (73% and 40% respectively) in a standard three dose immunization regimen given in two-week intervals. Delayed dosing combined with dose de-escalation elicited even greater inhibition in mice receiving MIP3α-CSP mRNA and CSP mRNA, with the MIP3α-CSP mRNA again demonstrating higher levels of inhibition (88% and 62% respectively). However, antibodies in the MIP3α-CSP group vs. CSP had relatively lower avidity for CSP. In passive transfer studies, higher avidity anti-CSP antibodies confer greater protection (66, 67). While antibody avidity to CSP and NANP may still be an important factor in protection, the greater relative and absolute numbers of antibodies to NANP in the MIP3α-CSP mRNA vaccinated mice in this study may have compensated for the reduced avidity of such antibodies. These findings suggest that the iDC-targeted vaccine formulation enhances protective immunity against malaria infection, potentially by promoting more robust and effective immune responses against the CSP protein on the sporozoite surface, thereby preventing hepatocyte invasion.

Fusion of the MIP3α cytokine to CSP also resulted in significantly greater TNFα, IL2, and IFNγ responses in CD4+ T cells. The proportion of CD4+ T cells producing TNFα, IL2, and IFNγ was 2–3x greater in the MIP3α-CSP vs. CSP vaccinated mice. Furthermore, with respect to IFNγ and TNFα, not only were the proportions of T cells that produced each cytokine increased in the MIP3α-CSP group, but also the splenocyte populations produced more of each cytokine, thereby amplifying cytokine effects. Interestingly, the proportion of polyfunctional CD4+ T cells was also increased following vaccination with the MIP3α-CSP mRNA vaccine. Polyfunctional T cells have an outsized role in vaccine efficacy with IL2, TNFα, and IFNγ showing synergism in their abilities to drive immune responses (75). Activated dendritic cells are highly associated with polyfunctional CD4+ T cell responses to malaria (76). We speculate that the iDC-targeting ability of the MIP3α-CSP mRNA elicits more polyfunctional CD4+ cells and T cell help, resulting in greater humoral responses.

The observed correlations between immune responses and protection from liver infection provide valuable insights into the mechanisms underlying CSP vaccine-induced immunity to malaria. Both polyfunctional CD4+ T cells and anti-NANP6 antibody titers correlated with protection, emphasizing the critical role of NANP-targeting antibodies and CD4+ T cells in preventing liver infection. Similar iDC-targeting approaches to potentiate T cell help may be utilized for other malaria vaccine candidates in a multi-stage mRNA vaccine against *P. falciparum*.
